# Adequate vitamin D status is associated with the reduced odds of prevalent diabetic retinopathy in African Americans and Caucasians

**DOI:** 10.1186/s12933-016-0434-1

**Published:** 2016-09-01

**Authors:** Amy E. Millen, Michelle W. Sahli, Jing Nie, Michael J. LaMonte, Pamela L. Lutsey, Barbara E. K. Klein, Julie A. Mares, Kirstin J. Meyers, Christopher A. Andrews, Ronald Klein

**Affiliations:** 1 Department of Epidemiology and Environmental Health, School of Public Health and Health Professions, University at Buffalo, The State University of New York, 270 Farber Hall, Buffalo, NY 14214-8001 USA; 2 Department of Public Health and Health Sciences, School of Health Professions and Studies, University of Michigan-Flint, Flint, MI USA; 3 Division of Epidemiology and Community Health, School of Public Health, University of Minnesota, Minneapolis, MN USA; 4 Department of Ophthalmology and Visual Sciences, School of Medicine and Public Health, The University of Wisconsin-Madison, Madison, WI USA; 5 Department of Ophthalmology and Visual Sciences, University of Michigan Medical School, Ann Arbor, MI USA

**Keywords:** Vitamin D, 25-hydroxy vitamin D, Diabetic retinopathy, Retinal diseases, Epidemiology, Cohort studies

## Abstract

**Background:**

Vitamin D status has been hypothesized to protect against development of diabetic retinopathy via its anti-inflammatory and anti-angiogenic properties. Additionally, in vitro and in vivo studies suggest vitamin D favorably influences blood pressure and blood glucose control, strong risk factors for diabetic retinopathy. We examined the association between vitamin D status and prevalent diabetic retinopathy in participants with diabetes from a population-based cohort.

**Methods:**

Among participants in the Atherosclerosis Risk in Communities (ARIC) study with diabetes at visit 3 (1993–1995), 1339 (906 Caucasians, 433 African Americans) had serum 25-hydroxyvitamin (25[OH]D) concentrations assessed at visit 2 (1989–1992) and nonmydriatic retinal photographs taken at visit 3. Dietary intake of vitamin D was assessed at visit 1 (1987–1989). Logistic regression was used to estimate odds ratios (ORs) and 95 % confidence intervals (CIs) for diabetic retinopathy by categories of season-adjusted 25(OH)D (<30 [referent], 30–<50, 50–<75 and ≥75 nmol/L), by quartile of vitamin D intake (IU/day), and use of vitamin D or fish oil supplements (yes/no). P for trend was estimated using continuous 25(OH)D or vitamin D intake. ORs were adjusted for race, and duration of diabetes. We further adjusted for HBA_1c_ and hypertension to examine if 25(OH)D influenced diabetic retinopathy via its effects on either glycemic control or blood pressure.

**Results:**

ORs (95 % CIs) for retinopathy, adjusted for race and duration, were 0.77 (0.45–1.32), 0.64 (0.37–1.10), and 0.39 (0.20–0.75), p for trend = 0.001, for participants with 25(OH)D of 30–<50, 50–<75, and ≥75 nmol/L, respectively. Further adjustment for hypertension minimally influenced results (data not show), but adjustment for HBA_1c_ attenuated the OR among those with 25(OH)D ≥75 (0.47 [0.23–0.96], p for trend = 0.030). No statistically significant association was observed between vitamin D intake from foods or supplements and retinopathy.

**Conclusions:**

25(OH)D concentrations ≥75 nmol/L were associated with lower odds of any retinopathy assessed 3 years later. We speculate this may be due in part to vitamin D’s influence on blood glucose control.

**Electronic supplementary material:**

The online version of this article (doi:10.1186/s12933-016-0434-1) contains supplementary material, which is available to authorized users.

## Background

Diabetic retinopathy is a leading cause of blindness in adults aged 20–74 years in the United States. Among individuals with diabetes it has direct influences on quality of life and functional independence of aging, affecting ~28.5 % of people with diabetes ≥40 years [1]. Modifiable nutritional factors may influence risk for diabetic retinopathy, but they have been relatively understudied in epidemiologic investigations [2]. Accumulating evidence from some [3–13], but not all [14–23], epidemiologic studies suggest that vitamin D status may be a novel modifiable risk factor for diabetic retinopathy.

Vitamin D status is hypothesized to affect risk for retinopathy [4] due to its immunomodulatory properties [24] as chronic low grade inflammation is hypothesized to promote the development of retinopathy [25]. Vitamin D is also hypothesized to positively regulate hypertension [26] and blood glucose control [27], both of which are strong risk factors for retinopathy [28, 29].

Using data from the prospective, population-based Atherosclerosis Risk in Communities (ARIC) Study, we investigated associations between vitamin D status, assessed with the blood biomarker of serum 25-hydroxyvitamn D (25[OH]D), and prevalent diabetic retinopathy assessed from graded fundus photographs taken 3 years later among Caucasian and African American participants with primarily type 2 diabetes (n = 1339). 25(OH)D reflects vitamin D from all sources (sunlight, diet and supplements). We hypothesized that individuals with higher 25(OH)D concentrations would have lower odds of retinopathy than participants with lower concentrations. We examined the extent to which this association was mediated by blood pressure or blood glucose control. We also explored associations between self-reported intake of vitamin D from foods and the odds of retinopathy.

## Methods

### Study sample

The ARIC Study, a population-based prospective study [30], recruited participants from Forsyth County, North Carolina; Jackson, Mississippi; the northwestern suburbs of Minneapolis, Minnesota; and Washington County, Maryland. Eligible participants were between 45 and 65 years of age at visit 1 (1987–1989) and intended to remain in the community in which they lived. All participants provided signed informed consent and the study protocol was approved by the institutional review boards at each ARIC study site and complies with the Helsinki Declaration as revised in 1983.

The present analyses use data collected at visits 1 (1987–1989), 2 (1990–1992) and 3 (1993–1995). This study sample consists of Caucasian and African American participants who were classified as having diabetes (fasting blood glucose of 126 mg/dl or non-fasting blood glucose of 200 mg/dl; self-report of a diabetes diagnosis; or use of medication for diabetes in the 2 weeks prior to the visit) at study visit 3, had gradable retinal fundus photos at visit 3 and serum 25(OH)D measures at visit 2. This was a retrospective analysis to examine the association between serum draw at visit 2, but recently (2012–2013) analyzed for 25(OH)D concentrations, and the prevalence of diabetic retinopathy determined 3 years later at visit 3.

There were 15,792 participants enrolled at visit 1, of which 12,887 attended visit 3. We excluded 796 participants who did not consent to use of their data to study outcomes other than cardiovascular disease. Of the remaining 12,091 participants, 1899 were classified as having diabetes of whom 350 were missing data on retinopathy status (301 missing retinal photos and 49 with upgradable photos), 186 were missing serum 25(OH)D, 8 identified as neither African American nor Caucasian, and 16 were missing data on pertinent covariates (glycosylated hemoglobin A_1c_ [HBA_1c_] or hypertension), providing a sample of 1339 participants. Analyses involving dietary vitamin D data had 1305 participants due to missing data on diet at visit 1.

### Retinal photography

Diabetic retinopathy was determined from grading of fundus photographs taken at visit three of one randomly selected eye. Participants sat in a dark room for 5 min to allow for nonpharmocological pupil dilution [31]. One 45-degree nonmydriatic retinal photograph was taken with a Canon CR-45UAF nonmydriatic film camera (Canon USA, Itasca, IL) and was centered to include the optic disc and the macula [31]. Fundus photographs were graded for the presence and severity of retinopathy at the University of Wisconsin Fundus Photograph Reading Center using a standard grading system, the modified Arlie House classification scheme [32]. Twenty-one percent (n = 280 of 1339) of participants had any retinopathy, of which 207 had mild non-proliferative diabetic retinopathy (NPDR), 44 had moderate to severe NPDR, 29 had proliferative diabetic retinopathy (PDR), and 3 had macular edema.

### Assessment of 25(OH)D

Vitamin D status was assessed by analyzing participants’ serum from fasting blood drawn at visit 2 for 25(OH)D concentrations (sum of 25[OH]D_2_ and 25[OH]D_3_) using liquid chromatography in tandem with high-sensitivity mass spectrometry (LC–MS) (Waters Alliance e2795; Waters, Milford, MA, USA) at the Collaborative Studies Clinical Laboratory at Fairview University Medical Center (Minneapolis, MN), as previously described [33]. Serum samples were stored at −80 °C from 1990–1992 until assessment of 25(OH)D from 2012 to 2013 [33]. The coefficient of variation, representing sample processing and laboratory error was 10.9 %. Differences in 25(OH)D concentrations due to season were accounted for using local regression [34]. 25(OH)D was regressed on day of blood draw and was conducted separately for Caucasians and African Americans. Residuals were added back to the sample mean (60.1 and 47.4 nmol/L for Caucasian and African Americans, respectively) and the season-adjusted values were used in all further mentioned analyses.

### Assessment of dietary and supplemental vitamin D intake

Dietary intake of vitamin D was assessed at visit 1 using a reliable and previously validated Willett 66-item semi-quantitative food frequency questionnaire (FFQ) [35, 36]. At visit 3, participants were asked about their use of vitamin D and fish oil supplements, as source of vitamin D. They were asked if they took fish oil (including omega-3 fatty acids, eicosapentaenoic acid [EPA], and cod liver oil), the duration of use, and the dose per week. Participants were also asked whether or not they took vitamin D “on a regular basis,” but no additional information was asked on duration of use or dose. There were 48 participants who reported use of either vitamin D or fish oil at visit 3.

### Assessment of additional participant characteristics

At each visit trained study personnel collected information on participants’ demographic factors, health history, family health history, smoking, medication use and other potential risk factors for cardiovascular disease [30]. Blood collected at visit 2 [37] was assessed for serum glucose, HBA_1c_ [38], hematocrit level [37], total plasma cholesterol, plasma triglyceride, low density lipoprotein (LDL), and high density lipoprotein (HDL) cholesterol concentrations [39].

Physical activity was assessed at visit 1 using a modified version [40] of the previously validated [41, 42] Baecke questionnaire from which we created a composite physical activity index score ranging from 0 (low overall physical activity) to 6. Duration of diabetes was defined as <3 years, 3 to <6, and ≥6 years determined using data on self-reported diabetes diagnosis, fasting and non-fasting blood glucose levels, and diabetes medication use collected at visits 1, 2 or 3. 25(OH)D concentrations and other covariate data used in these analyses were assessed at visit 2 with the exception of information on education, diet, physical activity (visit 1), and duration of diabetes (visit 3).

### Statistical analysis

Guided by the Institute of Medicine, vitamin D status was defined using 25(OH)D concentrations (nmol/L) as deficient (<30), inadequate (>30 to ≤50), and using two categories within the concentrations considered adequate (>50 to <75 and ≥75) [43]. Participant characteristics and risk factors for retinopathy were examined by vitamin D status, as well as by presence of retinopathy (any versus none), using t-tests, ANOVAs or Chi square tests.

Logistic regression was used to estimate the odds ratios (ORs) and 95 % confidence intervals (95 % CIs) for any prevalent retinopathy (both NPDR and PDR) by vitamin D status with the referent category of deficient status (<30 nmol/L) [43]. We also estimate the odds of having PDR or macular edema (n = 31) among participants with 25(OH)D ≥50 compared to <50 nmol/L. We had to apply the Firth bias-correction method for quasi-complete separation [44] due to the low number of outcomes. The ORs and 95 % CIs for retinopathy per 10 nmol/L difference in 25(OH)D are also presented and p-for trend analyses were conducted using 25(OH)D as a continuous variable.

Age, sex, race, education, duration of diabetes, smoking status, drinking status, ethanol intake, physical activity index score, body mass index (BMI), waist circumference, hematocrit level, LDL, HDL, total cholesterol and triglyceride concentrations were assessed as potential confounders of the vitamin D status and retinopathy association. If these variables were associated with either vitamin D status or prevalent retinopathy at a p value of 0.20 or less, we considered them for inclusion in the multivariable model. Using a forward, stepwise procedure, only potential confounders that changed the ORs ≥10 % were included in the adjusted model. The multivariable model was also adjusted for hypertension status and HBA_1c_ (as a measure of blood glucose control) to examine whether these variables mediated the 25(OH)D and retinopathy association.

A sensitivity analysis was conducted restricting our sample to include only individuals defined as having diabetes at both visits 2 (when 25[OH]D was measured) and 3. We wanted to examine if the association between vitamin D status and retinopathy would change when the sample was limited to those who were diagnosed with diabetes when 25(OH)D measures were assessed. Effect modification of the vitamin D and retinopathy association by age, sex, race, duration of diabetes and blood glucose control was explored by adding an interaction term to our logistic regression models. A p value <0.10 for the interaction term was considered statistically significant.

Variation in 25(OH)D concentrations explained by dietary intake of vitamin D was estimated using linear regression with season-adjusted 25(OH)D concentrations as the dependent variable and dietary vitamin D intake as the independent variable. Adjusted ORs and 95 % CIs for retinopathy in quartiles 2 through 4 (with quartile 1 as the referent) for dietary vitamin D intake (IU/day) and by category of reported frequency of consumption of vitamin D rich foods (never consumers as the referent) were estimated. A p for trend using continuous vitamin D intake or frequency of consumption, respectively, was estimated. We also estimated the odds of retinopathy in those who reported using vitamin D or fish oil supplements.

## Results

Seven percent of participants had deficient vitamin D status (25[OH]D < 30 nmol/L) and 59 and 16 % had adequate status with 25(OH)D concentrations ≥50 and ≥75 nmol/L, respectively (Table 1). Participants with adequate (≥75 nmol/L) compared to deficient vitamin D status were less likely to have retinopathy, be women, be from Jackson, MS, and have graduated high school, and they were more likely to be older and Caucasian. There was a greater proportion of former (compared to never or current) smokers with adequate versus deficient status. Individuals with adequate status had greater vitamin D intake, smaller waist circumferences, were less likely to be obese, and more likely to be physical active. On average their systolic blood pressure, HDL, glucose, and HBA_1c_ were lower, and their hematocrit and triglycerides were higher. Those with adequate status were also less likely to have used insulin in the last 2 weeks.

**Table 1:** Characteristics by vitamin D status of Caucasian and African American ARIC study participants classified as having diabetes, with gradable eye photo at visit 3 (1993–95), and available serum 25(OH)D concentrations at visit 2 (1990–92) (N = 1339)

	N	Vitamin D status defined by serum 25(OH)D concentrations (nmol/L)				p value	r (p value)^a^
		<30 deficient n = 96 (7 %)	30 to <50 Inadequate n = 454 (34 %)	50 to <75 Adequate n = 577 (43 %)	≥75 adequate n = 212 (16 %)		
Season-adjusted serum 25(OH)D, mean (SD)	1339	24.5 (4.9)	41.7 (5.5)	61.2 (6.9)	88.6 (15.2)	<0.001	NA
Prevalence of retinopathy, n (% yes)	1339	28 (29.2 %)	111 (24.4 %)	115 (19.9 %)	26 (12.3 %)	<0.001	^§^
Severity of retinopathy, n (%)						0.005	^§^
None	1059	68 (70.8 %)	343 (75.6 %)	462 (80.1 %)	186(87.7 %)		
Mild NPDR	207	22 (22.9 %)	82 (18.1 %)	88 (15.3 %)	15 (7.1 %)		
Moderate/severe NPDR	44	3 (3.1 %)	21 (4.6 %)	15 (2.6 %)	5 (2.4 %)		
Proliferative DR	29	3 (3.1 %)	8 (1.8 %)	12 (2.1 %)	6 (2.8 %)		
Demographics							
Age (years), mean (SD)	1339	56.4 (5.7)	57.0 (5.6)	57.7 (5.6)	57.9 (5.5)	0.046	0.08 (0.003)
Sex, n (% women)	1339	78 (81.3 %)	293 (64.5 %)	254 (44.0 %)	85 (40.1 %)	<0.001	^§^
Race, n (% Caucasians)	1339	38 (39.6 %)	249 (54.8 %)	428 (74.2 %)	191 (90.1 %)	<0.001	^§^
Field center, n (%)						<0.001	^§^
Forsyth County, NC	308	20 (20.8 %)	92 (20.3 %)	136 (23.6 %)	60 (28.3 %)		
Jackson, MS	374	50 (52.1 %)	168 (37.0 %)	137 (23.7 %)	19 (9.0 %)		
Minneapolis, MN	286	14 (14.6 %)	83 (18.3 %)	136 (23.6 %)	53 (25.0 %)		
Washington County, MD	371	12 (12.5 %)	111 (24.4 %)	168 (29.1 %)	80 (37.7 %)		
Education^b^, n (%)—visit 1						0.025	^§^
Basic or 0 years	370	25 (26.0 %)	126 (27.9 %)	154 (26.7 %)	65 (30.7 %)		
Intermediate	559	34 (35.4 %)	193 (42.8 %)	230 (39.9 %)	102 (48.1 %)		
Advanced	407	37 (38.5 %)	132 (29.3 %)	193 (33.4 %)	45 (21.2 %)		
Health and lifestyle characteristics							
Duration of diabetes, n (%)—visit 3						0.572	^§^
<3 years	300	26 (27.1 %)	89 (19.6 %)	131 (22.7 %)	54 (25.5 %)		
3 to <6 years	293	18 (18.8 %)	106 (23.3 %)	125 (21.7 %)	44 (20.8 %)		
≥6 years	746	52 (54.2 %)	259 (57.0 %)	321 (55.6 %)	114 (53.8 %)		
Smoking status, n (%)						0.034	^§^
Current	257	21 (21.9 %)	97 (21.5 %)	105 (18.2 %)	34 (16.0 %)		
Former	535	30 (31.3 %)	168 (37.2 %)	232 (40.2 %)	105 (49.5 %)		
Never	545	45 (46.9 %)	187 (41.4 %)	240 (41.6 %)	73 (34.4 %)		
Vitamin D intake (IU/day), mean (SD)—visit 1	1305	164.2 (117.3)	232.5 (154.4)	239.7 (151.7)	261.8 (147.4)	<0.001	0.16 (<0.001)
Vitamin D supplement, n (% yes)—visit 3	1330	0 (0.0 %)	8 (1.8 %)	9 (1.6 %)	6 (2.8 %)	0.352	^§^
Fish oil supplement use, n (% yes)—visit 3	1330	0 (0.0 %)	13 (2.9 %)	12 (2.1 %)	6 (2.8 %)	0.362	^§^
Drinking status, n (%)						0.056	^§^
Current	616	37 (38.5 %)	191 (42.2 %)	277 (48.0 %)	111 (52.4 %)		
Former	364	24 (25.0 %)	131 (28.9 %)	155 (26.9 %)	54 (25.5 %)		
Never	358	35 (36.5 %)	131 (28.9 %)	145 (25.1 %)	47 (22.2 %)		
Waist circumference (cm), mean (SD)	1337	112.2 (15.5)	109.2 (15.8)	106.9 (13.2)	102.9 (12.5)	<0.001	−0.16 (<0.001)
BMI category (kg/m^2^), n (%)						<0.001	−0.19 (<0.001)
Under/normal weight (<25 kg/m^2^)	164	6 (6.3 %)	45 (10.0 %)	70 (12.2 %)	43 (20.4 %)		
Overweight (25–30 kg/m^2^)	427	24 (25.0 %)	135 (29.9 %)	182 (31.6 %)	86 (40.8 %)		
Obese (≥30 kg/m^2^)	744	66 (68.8 %)	272 (60.2 %)	324 (56.3 %)	82 (38.9 %)		
Composite physical activity index—visit 1, mean (SD)	1335	2.2 (1.4)	2.7 (1.5)	3.0 (1.5)	3.3 (1.4)	<0.001	0.20 (<0.001)
Average diastolic blood pressure (mmHg), mean (SD)	1339	73.3 (9.3)	73.4 (11.3)	72.7 (10.3)	72.1 (9.5)	0.435	−0.03 (0.238)
Average systolic blood pressure (mmHg), mean (SD)	1339	127.9 (19.4)	129.0 (20.4)	126.0 (18.6)	125.1 (16.9)	0.028	−0.07 (0.008)
Hypertension^c^, n (% yes)	1339	56 (58.3 %)	258 (56.8 %)	317 (54.9 %)	107 (50.5 %)	0.424	^§^
Hematocrit (%), mean (SD)	1333	39.7 (4.2)	40.6 (3.8)	41.7 (3.7)	41.9 (3.4)	<0.001	0.17 (<0.001)
Total cholesterol (mg/dL), mean (SD)	1337	213.8 (38.0)	210.2 (40.5)	213.6 (41.9)	212.8 (42.5)	0.586	0.02 (0.406)
HDL (mg/dL), mean (SD)	1336	44.7 (12.0)	44.2 (14.4)	41.0 (13.4)	42.6 (14.2)	0.001	−0.09 (0.002)
LDL (mg/dL), mean (SD)	1279	138.8 (36.0)	133.2 (35.0)	135.5 (36.6)	133.9 (37.5)	0.504	0.01 (0.668)
Triglycerides (mg/dL), mean (SD)	1336	155.3 (82.2)	166.2 (98.3)	192.0 (132.0)	182.7 (104.9)	<0.001	0.08 (0.004)
Glucose (mg/dL), mean (SD)	1339	184.2 (89.5)	177.6 (76.6)	171.6 (74.7)	156.1 (62.3)	0.002	−0.11 (<0.001)
Glycosylated hemoglobin (%), mean (SD)	1339	7.8 (2.1)	7.7 (2.1)	7.4 (1.9)	6.9 (1.6)	<0.001	−0.14 (<0.001)
Insulin use in the past 2 weeks, n (% yes)	1339	16 (16.7 %)	81 (17.8 %)	70 (12.1 %)	25 (11.8 %)	0.039	^§^

Characteristics assessed at visit two unless otherwise noted

Individuals with diabetes were participants who had one of the following: (1) an 8 h fasting glucose ≥126 mg/dL, (2) a non-fasting glucose ≥200 mg/dL, (3) use of diabetes medication in the past 2 weeks, or (4) self-reported being told by a doctor that they had diabetes

^§^Correlation coefficient not presented because characteristic was not a continuous variable

^a^Spearman correlation coefficient and associated p value for the correlation between season-adjusted serum 25(OH)D and the respective continuous variable

^b^Education defined as Basic or 0 years (≤11 years or less, i.e., high school with no degree or less), Intermediate (12–16 years, i.e., high school graduate or vocational school), advanced (17–21 years, i.e., college or higher)

^c^Average systolic blood pressure ≥140 mm Hg, or diastolic ≥90 mm Hg, or high blood pressure medication use in the past 2 weeks

Of the 1339 diabetic participants, 21 % (n = 280) had DR. In crude analyses, individuals with 25(OH)D concentrations 50 to <75 and ≥75 nmol/L had lower odds of retinopathy than deficient individuals (Table 2). Only adjustment for race and duration of diabetes changed the odds ratio greater than 10 % and were included in the multivariable model. Adjustment for age or BMI, a strong predictor of 25(OH)D concentrations, had no additional influence on the model and thus was not adjusted for in these analyses. After adjustment for these covariates there was a significant 61 % lower odds of retinopathy for those with 25(OH)D concentrations ≥75 nmol/L, with a significant p for trend of 0.001 and a 13 % lower odds of retinopathy with each additional 10 nmol/L in serum 25(OH)D concentrations. Further adjustment for HBA_1c_ attenuated the association, but did not remove statistical significance. The odds of participants having proliferative diabetic retinopathy or macular edema among those with 25(OH)D ≥50 nmol/L (19 out of 789 at risk) compared to those with 25(OH)D <50 nmol/L (12 out of 550 at risk) was 1.48 (0.70–3.12) adjusted for race, duration, HBA_1c_ and hypertension status. The adjusted odds ratio per 10 nmol/L difference in 25(OH)D was 1.07 (0.89–1.29), p for trend =0.473.

**Table 2:** Crude and adjusted OR and 95 % CIs for the diabetic retinopathy by vitamin D status among Caucasian and African American ARIC study participants classified as having diabetes and having gradable eye photos at visit 3 (1993–95), and available serum 25(OH)D concentrations at visit 2 (1990–92) (N = 1339)

Model	Vitamin D status defined by serum 25(OH)D concentrations (nmol/L)					
	<30 deficient	30 to <50 inadequate	50 to <75 adequate	≥75 adequate	p trend*	Continuous, per 10 nmol/L
#With retinopathy/#in category	28/96	111/454	115/577	26/212		
Crude model	1	0.79 (0.48–1.28)	0.61 (0.37–0.98)	0.34 (0.19–0.62)	<0.001	0.85 (0.79–0.92)
Model 1^a^	1	0.77 (0.45–1.32)	0.64 (0.37–1.10)	0.39 (0.20–0.75)	0.001	0.87 (0.81–0.95)
Model 1 + HBA_1c_^b^	1	0.81 (0.45–1.45)	0.70 (0.39–1.27)	0.47 (0.23–0.96)	0.030	0.91 (0.83–0.99)
Model 1 + hypertension status^b^	1	0.77 (0.45–1.32)	0.63 (0.37–1.09)	0.38 (0.20–0.75)	0.001	0.87 (0.81–0.95)
Model 1 + HBA_1c_ + hypertension status	1	0.81 (0.45–1.46)	0.70 (0.39–1.25)	0.47 (0.23–0.96)	0.026	0.91 (0.83–0.99)

* p for trend calculated using season adjusted serum 25(OH)D as a continuous variable

^a^Model 1: adjusted for race and duration of diabetes

^b^HBA_1c_ was entered as a continuous variable; hypertension status is defined as in Table 1

The observed lower odds of retinopathy among participants with adequate compared to deficient vitamin D status remained regardless of age, sex, race, duration of diabetes and glycemic control, except for observations in the youngest age group (54 years and younger) (Table 3). There were not statistically significant interactions. A sensitivity analysis removing participants who were not classified as having diabetes at visit 2 (n = 336), when 25(OH)D concentrations were measured, did not substantially change the main findings. The odds of retinopathy in participants with 25(OH)D ≥75 compared to <30 nmol/L was 0.43 (0.21–0.88), p for trend = 0.005 after adjustment for race and duration and 0.54 (0.25–1.15), p for trend = 0.055 with further adjustment for HBA_1c_ and hypertension status.

**Table 3:** Adjusted OR and 95 % CIs for diabetic retinopathy by vitamin D status stratified by age, sex, race, duration of diabetes, and HbA1c levels among Caucasian and African American ARIC study participants classified as having diabetes, with gradable eye photo at visit 3 (1993–95), and available serum 25(OH)D concentrations at visit 2 (1990–92) (N = 1339)

	Vitamin D status assessed with serum 25(OH)D concentrations (nmol/L)					
	<30 deficient	30 to <50 inadequate	50 to <75 adequate	≥75 adequate	p trend*	Continuous, per 10 nmol/L
Age group						
47 to 54 years (n = 471)						
#with DR/# in group	8/40	40/172	33/195	10/64		
Adjusted OR (95 % CI)^a^	1	1.20 (0.42–3.44)	0.90 (0.31–2.64)	1.44 (0.39–5.25)	0.800	1.02 (0.87–1.20)
55–59 years (n = 356)						
#with DR/# in group	6/26	24/124	30/147	7/59		
Adjusted OR (95 % CI)	1	0.84 (0.23–3.04)	1.12 (0.31–4.08)	0.88 (0.19–4.07)	0.686	0.96 (0.80–1.16)
60–64 years (n = 332)						
#with DR/# in group	9/19	30/103	35/149	5/61		
Adjusted OR (95 % CI)	1	0.43 (0.14–1.36)	0.39 (0.12–1.20)	0.10 (0.02–0.45)	0.011	0.80 (0.68–0.95)
65 to 68 years (n = 180)						
#with DR/# in group	5/11	17/55	17/86	4/28		
Adjusted OR (95 % CI)	1	0.64 (0.13–3.09)	0.38 (0.08–1.81)	0.27 (0.04–1.69)	0.203	0.86 (0.68–1.08)
p for interaction	0.372					
Sex						
Men (n = 629)						
#with DR/# in group	5/18	38/161	64/323	12/127		
Adjusted OR (95 % CI)	1	0.67 (0.19–2.36)	0.52 (0.15–1.79)	0.23 (0.06–0.89)	0.019	0.85 (0.75–0.97)
Women (n = 710)						
#with DR/# in group	23/78	73/293	51/254	14/85		
Adjusted OR (95 % CI)	1	0.80 (0.41–1.59)	0.68 (0.33–1.38)	0.78 (0.31–1.97)	0.262	0.93 (0.82–1.05)
p for interaction	0.320					
Race						
Caucasian (n = 906)						
#with DR/# in group	10/38	55/249	73/428	23/191		
Adjusted OR (95 % CI)	1	0.72 (0.29–1.81)	0.52 (0.21–1.28)	0.40 (0.15–1.07)	0.072	0.91 (0.82–1.01)
African American (n = 433)						
#with DR/# in group	18/58	56/205	42/149	3/21		
Adjusted OR (95 % CI)	1	0.89 (0.42–1.89)	0.98 (0.45–2.16)	0.45 (0.10–2.15)	0.268	0.91 (0.77–1.08)
p for interaction	0.555					
Duration of diabetes,						
<*6* *years (n* = *593)*						
#with DR/# in group	5/44	13/195	15/256	1/98		
Adjusted OR (95 % CI)	1	0.53 (0.17–1.60)	0.48 (0.16–1.45)	0.08 (0.01–0.72)	0.014	0.77 (0.62–0.95)
≥*6* *years (n* = *746)*						
#with DR/# in group	23/52	98/259	100/321	25/114		
Adjusted OR (95 % CI)	1	0.99 (0.50–1.95)	0.84 (0.43–1.65)	0.68 (0.30–1.52)	0.219	0.94 (0.85–1.04)
p for interaction	0.417					
HBA_1c_ levels						
≤*7* *% (adequate control) (n* = *756)*						
#with DR/# in group	5/47	21/241	16/326	4/142		
Adjusted OR (95 % CI)	1	0.75 (0.26–2.13)	0.44 (0.15–1.29)	0.26 (0.06–1.07)	0.091	0.86 (0.73–1.02)
>*7* *% (inadequate control) (n* = *583)*						
#with DR/# in group	23/49	90/213	99/251	22/70		
Adjusted OR (95 % CI)	1	0.93 (0.47–1.83)	0.89 (0.45–1.77)	0.65 (0.28–1.51)	0.163	0.93 (0.83–1.03)
p for interaction	0.290					

* p for trend calculated using serum 25(OH)D as a continuous variable

^a^Model adjusted for race, duration of diabetes, HbA1c (continuous), and hypertension status. Strata of HbA1c are further adjusted for continuous levels of HBA_1c_

Dietary vitamin D intake of vitamin D from foods accounted for 1 % of the between person variation in 25(OH)D concentrations in this sample. No statistically significant associations were found between vitamin D intake from foods and retinopathy (Additional file 1: Table S1). Intake of 1 serving (3–5 oz) of dark fish >1/week compared to never was associated with a 68 % lower odds of retinopathy with a p for continuous trend of 0.060. Further adjustment by intake of omega-3 polyunsaturated fatty acids (PUFAs) did not attenuate this association (data not shown). The odds of retinopathy among vitamin D and fish oil supplement users compared to nonusers was 0.63 (0.25–1.64) with adjustment for race, duration of diabetes, HBA_1c_, and hypertension status.

## Discussion

We observed a dose–response association between 25(OH)D concentrations and diabetic retinopathy, suggesting that individuals with higher 25(OH)D concentrations have lower odds of prevalent retinopathy, primarily NPDR. No statistically significant association was observed between 25(OH)D and severe disease (PDR or macular edema) although the number of cases was small (n = 31). A protective association with intake of vitamin D from all foods combined was not observed. Assessment of dietary vitamin D intake, as measured, does not likely reflect or enhance vitamin D status as we found vitamin D intake only explained a minimal amount of the between person variation in 25(OH)D concentrations in this sample. We did observe that frequent consumption (>1 time per week) of dark fish compared to never eating this type of fish was associated with a decreased odds for retinopathy. Fish are a rich source of vitamin D as well as omega-3 PUFAs (eicosapentaenoic and docosahexaenoice acid). omega-3 have anti-inflammatory properties [45], but adjustment for intake of omega-3 PUFAs did not confound this association.

Previous research on the association between vitamin D status and diabetic retinopathy has predominantly focused on samples of individuals with type 2 diabetes [3–8, 11–17, 19, 21–23], similar to ARIC, with some research focused on individuals with type 1 diabetes [9, 10, 18, 20]. A number of studies have compared 25(OH)D concentrations between groups of individuals with and without diabetic retinopathy in case–control designs [3, 11, 13, 17, 22] with a protective association of 25(OH)D on prevalent retinopathy found in three studies [3, 11, 13]. The majority of other studies consist of cross-sectional designs recruiting participants from clinical settings [5, 6, 9, 10, 12, 14, 15, 19, 21, 23] with half of these studies supporting a protective association of vitamin D with retinopathy [5, 6, 9, 10, 12]. All noted studies recruited participants with diabetes from clinic settings, perhaps limiting the generalizability of study findings. Other limitations include small sample sizes (n ≤ 300 for samples of individuals with diabetes) [5, 10, 11, 13–15, 17, 23], lack of multivariate adjusted analysis [14, 17], inclusion of strong determinants of 25(OH)D concentrations in multivariable models which may result in overadjustment [20], and assessment of retinopathy status from ophthalmologist examination rather than from standardized grading of retinal fundus photographs [3, 5, 11–15, 17, 19, 21–23].

Results from nationally representative surveys [4, 6] comprised primarily of individuals with type 2 diabetes have supported a protective association between retinopathy status and 25(OH)D concentrations; however a population-based cohort [20] of individuals with type 1 diabetes has not. Strengths of these studies include the use of graded, retinal photographs, adjustment for other confounding factors, and large sample sizes (~500 + participants). These cross-sectional studies cannot establish temporality of the vitamin D and retinopathy association, similar to the present study.

Only three studies to date have examined prospective associations between vitamin D status and risk of retinopathy [8, 16, 18]. No statistically significant association was observed between 25(OH)D concentrations and the 26-year incidence of either background or proliferative retinopathy among 220 patients with type 1 diabetes attending a diabetes center [18] or with the 5-year incidence or progression of retinopathy in the Veterans Affairs Diabetes Trial (n = 955) [16]. A recent study of 9524 participants with type 2 diabetes from the Fenofibrate intervention and Event Lowering Diabetes (FIELD) Trial were followed for development microvascular complications, including retinopathy determined by on-study laser treatment (not fundus photography). [8] They observed a significant 13 % (p = 0.03) lower odds of microvascular complications with each baseline 50 nmol/L difference in 25(OH)D. Further adjustment of the multivariable model for HBA_1c_, physical activity or seasonal variability attenuated the association and removed its statistical significance. In our study, the association between vitamin D status and retinopathy was also attenuated after adjustment for glycemic control. It is unclear whether adjustment for HBA_1c_ confounds the observed association or results in over adjustment because vitamin D protects against retinopathy via its influence on glycemic control.

Vitamin D is proposed to have a role in ocular health. Expression of the vitamin D receptor (VDR) in the retina [46] and in human cultured retinal endothelial cells [47], support this hypothesis. Further, the enzyme 1-α-hydroxylase, responsible for synthesis of 1,25(OH)_2_D, is expressed in the retina suggesting a local action of the hormone calcitriol (1,25(OH)_2_D) in the eye [46].

In vitro studies [48] and animal models of diabetes [49] suggest that chronic low grade inflammation plays a role in the development of diabetic retinopathy; however, evidence of associations between biomarkers of systemic inflammation and diabetic retinopathy in epidemiologic studies still remains inconclusive [50]. High blood glucose is thought to increase adhesion of leukocytes to microvascular endothelial cells leading to cell damage, impaired blood flow [49, 51], and consequential retinopathy lesions [52, 53]. We hypothesize that vitamin D may down-regulate a localized, ocular, pro-inflammatory state by suppressing pro-inflammatory cytokines and other toxic agents [24]. This is supported by a study in cultured endothelial cells showing that vitamin D reduces the damaging effects of advanced glycation end products [54].

The VDR is expressed in human pancreatic beta-cells [55] and the human insulin receptor gene’s promoter has a vitamin D response element [56], suggesting a possible role in blood glucose control. To date, in vitro cell culture and animal model studies of diabetes examining the effect of 1,25(OH)_2_D on beta cell function, insulin receptor gene expression, and glucose uptake are inconclusive [57]. A recent meta-analysis suggests no association between randomized controlled vitamin D supplementation trials and glucose homeostasis or diabetes prevention; however, this study could not make conclusions with respect to the effect of long-term supplementation and micro- or macro-vascular complications of diabetes [58].

Our study is limited by its cross-sectional design and therefore cannot determine the temporality of this association between vitamin D and retinopathy. We are also limited by the availability of retinal photographs taken of one field from only one eye. There may be misclassification of endpoints ascertained at visit 3. However, as the photographed eye was chosen randomly, we would expect nondifferential misclassification of our endpoint which would bias our observed risk estimates toward the null. We also could not adequately explore the association between vitamin D and proliferative retinopathy due to the small number of participants with this outcome. Vitamin D has been shown to inhibit angiogenesis in an animal model of oxygen-induced ischemic retinopathy [59] and inhibit vascular endothelial growth factor and transforming growth factor-β_1_ expression in retinal tissues of experimentally induced diabetes in rats [60]. We also did not have data on sunlight exposure, and thus were unable to examine the association between vitamin D and diabetic retinopathy inclusive of all relevant sources contributing to circulating 25(OH)D concentrations.

Our study’s strength include a well-defined population of individuals with diabetes and availability of numerous, measured covariates that we could adjust for as potential confounding factors, although we realize that residual confounding may exist. Our study was population-based and is most generalizable to individuals with type 2 diabetes who comprised the majority of our sample. We were able to examine this association in both Caucasians and African Americans, showing that associations did not vary by race. We had retinal photographs, graded in a standardized fashion, to assess retinopathy and 25(OH)D and assessed using LC–MS, the gold standard for vitamin D assessment [61], with standardized, quality control measures taken. Our study contributes to the body of evidence supporting a protective, association between 25(OH)D and prevalent diabetic retinopathy that is consistent across racial groups.

## Conclusions

In conclusion, adequate vitamin D status, 25(OH)D concentrations ≥75 nmol/L, may be associated with reduced odds of diabetic retinopathy. We speculate that the influence of vitamin D on diabetic retinopathy may be, in part, via its influence on blood glucose control.

## Additional file

10.1186/s12933-016-0434-1 Adjusted* ORs and 95 % CI for diabetic retinopathy by reported quartile (Q) of dietary vitamin D intake from foods (IU/day) and by frequency of consumption of vitamin D rich foods at visit 1 (1987–1989) among Caucasian and African American ARIC study participants classified as having diabetes and having gradable eye photos at visit 3 (1993–95) and dietary data at visit 1 (N = 1305^†^).

## Abbreviations

25[OH]D: 25-hydroxy vitamin D
ARIC: atherosclerosis risk in communities study
BMI: body mass index
DR: diabetic retinopathy
HBA_1c_: glycosylated hemoglobin A_1c_
HDL: high density lipoprotein
LDL: low density lipoprotein
NPDR: non-proliferative diabetic retinopathy
PDR: proliferative diabetic retinopathy
VDR: vitamin D receptor
