# Supplementary material for: Adequate vitamin D status is associated with the reduced odds of prevalent diabetic retinopathy in African Americans and Caucasians
Source: Cardiovasc Diabetol. 2016 Sep 1;15:128. doi: 10.1186/s12933-016-0434-1 (PMC5009647; doi:10.1186/s12933-016-0434-1)
Supplement: Supplementary file 1 — 10.1186/s12933-016-0434-1 Adjusted* ORs and 95 % CI for diabetic retinopathy by reported quartile (Q) of dietary vitamin D intake from foods (IU/day) and by frequency of consumption of vitamin D rich foods at visit 1 (1987–1989) among Caucasian and African American ARIC study participants classified as having diabetes and having gradable eye photos at visit 3 (1993–95) and dietary data at visit 1 (N = 1305†). [file 12933_2016_434_MOESM1_ESM.doc]

**Additional file 1: Table S1.**

| **Supplemental Table 1**: Adjusted***** ORs and 95%CI for diabetic retinopathy by reported quartile (Q) of dietary vitamin D intake from foods (IU/day) and by frequency of consumption of vitamin D rich foods at visit 1 (1987-1989) among Caucasian and African American ARIC study participants classified as having diabetes and having gradable eye photos at visit 3 (1993-95) and dietary data at visit 1 **(N=1305** ^†^**)** | | | | | |
| --- | --- | --- | --- | --- | --- |
|  |  | | | |  |
|  | **Category of selected food intake by frequency of consumption** | | | | **p-trend**^‡^ |
|  |  |  |  |  |  |
| **Vitamin D intake (Q):** **(range)** | **Q1: (11.2 - 132.8)** | **Q2: (132.9 -203.5)** | **Q3: (203.5 - 300.5)** | **Q4: (301.0 - 1041.5)** |  |
| # with DR / # in group | 58/326 | 69/326 | 77/327 | 70/326 |  |
| Adjusted OR (95% CI) | 1 | 1.38 (0.88-2.17) | 1.56 (1.00-2.44) | 1.20 (0.76-1.89) | 0.740 |
|  |  |  |  |  |  |
| **Skim or low fat milk (8 oz.)** | **Never** | **1/month to <1/day** | **1/day** | **>1/day** |  |
| # with DR / # in group | 85/482 | 67/289 | 89/364 | 33/170 |  |
| Adjusted OR (95% CI) | 1 | 1.65 (1.08-2.51) | 1.72 (1.15-2.57) | 1.13 (0.67-1.91) | 0.596 |
|  |  |  |  |  |  |
| **Whole milk (8 oz.)** | **Never** | **1/month to <1/day** | **1/day** | **>1/day** |  |
| # with DR / # in group | 185/880 | 59/294 | 22/95 | 8/36 |  |
| Adjusted OR (95% CI) | 1 | 1.01 (0.69-1.49) | 1.44 (0.80-2.57) | 0.88 (0.35-2.23) | 0.434 |
|  |  |  |  |  |  |
| **Dark fish (3 to 5 oz.)**^§^ | **Never** | **1/month to <1/week** | **1/week** | **>1/week** |  |
| # with DR / # in group | 139/687 | 85/374 | 41/188 | 9/56 |  |
| Adjusted OR (95% CI) | 1 | 1.00 (0.70-1.43) | 0.95 (0.60-1.51) | 0.32 (0.14-0.78) | 0.060 |
|  |  |  |  |  |  |
| **Other fish (3 to 5 oz.)**^§^ | **Never** | **1/month to <1/week** | **1/week** | **>1/week** |  |
| # with DR / # in group | 75/365 | 77/407 | 76/356 | 46/177 |  |
| Adjusted OR (95% CI) | 1 | 0.76 (0.50-1.16) | 0.80 (0.52-1.25) | 1.16 (0.70-1.92) | 0.638 |
|  |  |  |  |  |  |
| **^*^** Odds ratios were adjusted for race, duration of diabetes, HBA_1c_ (continuous), and hypertension status.  ^†^ There were 34 participants of the 1,339 with missing dietary vitamin D data at visit 1  ^‡^p for trend was calculated using dietary vitamin D intake or frequency of consumption of selected food at visit 1 as a continuous variable.  ^§^ Dark meat fish such as salmon, mackerel, swordfish, sardines, bluefish; other fish, such as cod, perch, catfish, etc. | | | | | |
